# A combination of two antibodies recognizing non‐overlapping epitopes of HER2 induces kinase activity‐dependent internalization of HER2

**DOI:** 10.1111/jcmm.12899

**Published:** 2016-07-28

**Authors:** Monika Szymanska, Anne M. Fosdahl, Filip Nikolaysen, Mikkel W. Pedersen, Michael M. Grandal, Espen Stang, Vibeke Bertelsen

**Affiliations:** ^1^ Department of PathologyOslo University HospitalOsloNorway; ^2^ Institute of Clinical MedicineUniversity of OsloOsloNorway; ^3^ Symphogen A/SBallerupDenmark

**Keywords:** HER2/ErbB2, monoclonal antibodies, antibody combinations, kinase activity, endocytosis, degradation

## Abstract

The human epidermal growth factor receptor 2 (HER2/ErbB2) is overexpressed in a number of human cancers. HER2 is the preferred heterodimerization partner for other epidermal growth factor receptor (EGFR) family members and is considered to be resistant to endocytic down‐regulation, properties which both contribute to the high oncogenic potential of HER2. Antibodies targeting members of the EGFR family are powerful tools in cancer treatment and can function by blocking ligand binding, preventing receptor dimerization, inhibiting receptor activation and/or inducing receptor internalization and degradation. With respect to antibody‐induced endocytosis of HER2, various results are reported, and the effect seems to depend on the HER2 expression level and whether antibodies are given as individual antibodies or as mixtures of two or more. In this study, the effect of a mixture of two monoclonal antibodies against non‐overlapping epitopes of HER2 was investigated with respect to localization and stability of HER2. Individual antibodies had limited effect, but the combination of antibodies induced internalization and degradation of HER2 by multiple endocytic pathways. In addition, HER2 was phosphorylated and ubiquitinated upon incubation with the antibody combination, and the HER2 kinase activity was found to be instrumental in antibody‐induced HER2 down‐regulation.

## Introduction

HER2/ErbB2 belongs to the ErbB‐ or epidermal growth factor receptor (EGFR) family of receptor tyrosine kinases, which in addition consists of EGFR/HER1, ErbB3/HER3 and ErbB4/HER4. Except for HER2, the receptors bind a variety of ligands. Ligand binding induces conformational changes which favour receptor dimerization and activation of the kinase domains, and both homo‐ and heterodimers can be formed. No ligand has been identified for HER2, but the receptor does constitutively adopt an open conformation, similar to the ligand‐bound conformation of the other receptors, making HER2 the preferred heterodimerization partner (reviewed in 1). As a result of the various physical properties of the ligands and the variety of dimers that can be formed, activation of ErbB proteins regulates both cellular growth and differentiation, as well as proliferation, migration and survival. A tight regulation is thus important, and mutations, gene amplifications and/or overexpression of ErbB proteins are associated with a number of human malignancies. HER2 is amplified and/or overexpressed in cancers such as breast, colorectal, gastric and lung and is often associated with poor prognosis (reviewed in 2).

One way by which cells regulate ErbB‐mediated signalling is through ligand‐induced internalization followed by receptor inactivation or trafficking to late endosomes and lysosomes for degradation. EGFR and ErbB3 are both internalized *via* clathrin‐dependent endocytosis, but in addition to clathrin‐mediated internalization, also several clathrin‐independent pathways exist (for a recent review see 3). In contrast to EGFR and ErbB3, HER2 is endocytosis impaired 4, 5 and HER2‐containing dimers are either retained at the plasma membrane or very efficiently recycled upon internalization (reviewed in 1, 6, 7).

Different strategies have been developed to target HER2. HER2 is stabilized through interaction with Heat shock protein 90 (Hsp90). Heat shock protein 90 inhibition induces internalization and degradation of HER2 and a number of Hsp90 inhibitors are in clinical trials. Another strategy is the use of kinase inhibitors, such as lapatinib (Tyrkerb) and afatinib (Gilotrif), which are both in clinical use. In addition, antibodies have become important therapeutic tools in treatment of HER2 overexpressing tumours (for recent reviews see 8, 9, 10, 11, 12). The mechanisms of action of therapeutic antibodies are complex. In the clinical setting antibody‐dependent cellular cytotoxicity (ADCC) is important, but antibodies do in addition have other important functions such as inhibition of receptor dimerization and activation, and induction of internalization and down‐regulation of receptors. However, the molecular mechanisms involved in antibody‐induced internalization of HER2 have only to a limited degree been identified. Previous studies have shown that incubation with anti‐HER2 antibodies can induce HER2 ubiquitination 13, 14, 15, 16, and ubiquitination is an important signal for internalization and degradation. Although HER2 internalization can be induced by single antibodies 13, most studies conclude that a combination of a minimum of two antibodies with non‐competitive binding sites is needed for efficient internalization 16, 17, 18, 19. A likely explanation is that whereas a single antibody only cross‐links two HER2 molecules, the combination of antibodies with non‐competing binding sites can induce an extensive cross‐linking. Such a clustering may lead to increased receptor down‐regulation not only by induction of internalization, but also by inhibited recycling as reported for antibody‐induced down‐regulation of EGFR 17, 20. Using a similar approach we recently characterized antibody‐induced internalization and degradation of EGFR which was found to occur by clathrin‐ and dynamin‐independent macropinocytosis 21.

In a recent study a number of new anti‐HER2 antibodies was characterized with respect to anti‐tumour activity, proliferation, cell‐cycle arrest and cell death, and, among others, HER2 activation, internalization and degradation 19. The combination of two or three antibodies binding to distinct domains of HER2 showed in all aspects superior efficiency compared with single antibodies. In this study, the mechanisms controlling antibody‐induced internalization and degradation of HER2 was investigated in details for the most efficient antibody pair identified in the study by Pedersen *et al*. 19.

## Materials and methods

### Materials

Green fluorecent protein (GFP)‐tagged dynamin 2 K44A was from Addgene (Cambridge, MA, USA) and GFP‐tagged CHIP was a generous gift from Cam Patterson (New York Presbyterian Hospital, NY, USA). Lapatinib was from Abcam plc (Cambridge, UK) and 17‐AAG was from Tocris Bioscience (Bristol, UK). Amiloride was from Santa Cruz Biotechnology, Inc. (Santa Cruz, CA, USA). Unless noted, all other materials and chemicals were from Sigma‐Aldrich (St. Louis, MO, USA).

### Antibodies

Anti‐HER2 mouse‐human chimeric antibodies mAb 4517, mAb 4384 19 and a negative control antibody were from Symphogen A/S (Ballerup, Denmark). The negative control antibody is of human origin, targets a non‐mammalian antigen and has been confirmed not to bind HER2 (Fig. S1). Mouse anti‐HER2 extracellular domain (clone 42) antibody was from BD Biosciences (Erembodegem, Belgium). Mouse anti‐HER2 extracellular domain (clone TAB 250), rabbit anti‐HER2 intracellular domain (clone Z4881), Alexa Fluor 555‐conjugated donkey anti‐goat and anti‐rabbit IgG antibodies were from Life Technologies (Carlsbad, CA, USA). Rabbit anti‐HER2 intracellular domain (29D8), anti‐phosphorylated Erk1/2 (T202/204) and anti‐phosphorylated Akt (S473) antibodies were from Cell Signalling Technology, Inc. (Danvers, MA, USA). Mouse anti‐HER2 intracellular domain (Ab‐3) was from Merck Millipore (Darmstadt, Germany). Rabbit anti‐lysosome‐associated membrane protein 1 (Lamp1) antibody was from Abcam plc. Mouse anti‐ubiquitin, anti‐phosphorylated tyrosine (pY), anti‐HER2 pY1112 (19G5), anti‐Hsp70 (W27), rabbit anti‐β‐tubulin, anti‐Erk1, goat anti‐early endosome antigen 1 (EEA1) and anti‐Akt1 antibodies were from Santa Cruz Biotechnology, Inc. Rabbit anti‐mouse IgG antibody was from Cappel Research Reagents (ICN Biochemicals, Irvin, CA, USA). Mouse anti‐GFP (11814 460001) antibody was from Roche (Mannheim, Germany). Rabbit anti‐human IgG, Alexa Fluor 488‐conjugated donkey anti‐mouse IgG, DyLight 488‐conjugated donkey anti‐rabbit IgG, Rhodamine Red‐X‐conjugated donkey anti‐human IgG and peroxidase‐conjugated donkey anti‐rabbit, anti‐sheep and anti‐mouse IgG antibodies were from Jackson Immuno‐Research Laboratories Inc (West Grove, PA, USA).

### Cell lines

OE19 cells from German Collection of Microorganisms and Cell Cultures (Braunschweig, Germany) and NCI‐N87 cells from the American Tissue Culture Collection (ATCC; Manassas, VA, USA) were grown in RPMI‐1640 with L‐glutamine and NaHCO_3_, supplemented with 100 units of Potassium Penicillin and 100 μg of Streptomycin Sulfate per ml media (Lonza Group Ltd., Basel, Switzerland) and 10% (v/v) fetal bovine serum (FBS). SK‐BR‐3 cells, from ATCC, were grown in DMEM high Glucose with UltraGlutamine (Lonza Group Ltd.) and supplemented with 100 units of Potassium Penicillin and 100 μg of Streptomycin Sulfate per ml and 15% (v/v) FBS.

### Incubation with antibodies

Cells were incubated with single antibodies (mAb 4517, mAb 4384 or negative control antibody) or the mAb mixture (mAb 4517 + mAb 4384) diluted to a final concentration of 25 μg/ml in complete growth medium or MEM containing 0.1% bovine serum albumin. All incubations with antibodies as well as pre‐incubations with the different reagents/inhibitors were performed at 37°C for the indicated time points.

### Transfection of cells

For knock‐down experiments, cells were transfected with short interfering RNA (siRNA) twice with a 48 hr interval using *Trans*IT‐X2^®^ (Mirus Bio LLC, Madison, WI, USA) according to manufacturers recommendations. For siRNA‐mediated knock down of Cbl, a combination of Cbl‐b (sc 29950) and c‐Cbl (sc 29242) siRNA, both from Santa Cruz Biotechnology, Inc., was used. For knock down of clathrin heavy chain, siRNA duplex to the target sequence GCAAUGAGCUGUUUGAAGA 22 was synthesized and annealed by Life Technologies. On‐TARGET plus Non‐targeting siRNA #2 (D‐001810‐02) was from GE Healthcare (Buckinghamshire, UK). For transient transfection with a plasmid encoding GFP‐tagged dynamin 2 K44A or GFP‐tagged CHIP, OE19 cells were seeded 1 day prior to transfection. Cells were transfected with plasmid using 1.5 μg DNA/ml Opti‐MEM^®^ (Life Technologies) with *Trans*IT‐X2^®^ according to manufacturers recommendations and incubated for 5 hrs. The medium was replaced with RPMI with 2 mM L‐glutamine and 10% (v/v) FBS and the cells were further incubated for 20 hrs before analysis.

### Immunocytochemistry and confocal microscopy

Cells were grown on 10 × 10 mm High Precision glass cover slips (Paul Marienfeld GmbH & Co. KG, Lauda‐Königshofen, Germany) and incubated with indicated antibodies and reagents as described in figure legends. After incubation, cells were washed three times with PBS and fixed in pre‐warmed (37°C) 10% Neutral Buffered Formalin (4% w/v formaldehyde) solution for 10 min. at room temperature and immunostained as described previously 21. To enhance the GFP signal, GFP‐booster Atto 488 (ChromoTek Inc., Hauppauge, NY, USA) was added together with secondary antibodies. Samples were mounted by ProLong Gold (Life Technologies) and images were acquired by Olympus FV1000 (Olympus Corporation, Tokyo, Japan). Pictures were processed with ImageJ software (National Institutes of Health, Bethesda, MD, USA) and Adobe Photoshop (Adobe Systems Inc., San Jose, CA, USA).

### Immunoblotting

Cells were lysed on ice in SDS lysis buffer [10 mM Tris, pH 6.8, 5 mM ethylenediaminetetraacetic acid (EDTA), 50 mM NaF, 30 mM sodium pyrophosphate, 2% SDS] supplemented with 250 μg/ml of AEBSF (A8456) and 1:100 (v/v) of Phosphatase inhibitor cocktail (P5726). Cell lysates were then homogenized with QIAshredder (Qiagen, Valencia, CA, USA), and incubated for 10 min. at 95°C in the presence of sample buffer (80% glycerol, 20% β‐Mercaptoethanol, 0.03% Bromophenol Blue). Proteins were subjected to SDS‐gel electrophoresis and protein blotting with 10% Mini‐PROTEAN^®^TGX^™^ Precast Gels and Trans‐Blot^®^ Turbo^™^ Transfer System (Bio‐Rad Laboratories, Hercules, CA, USA). The membranes were incubated with primary and secondary antibodies diluted in 1% Blotting‐Grade Blocker (Bio‐Rad Laboratories) for 1 hr at room temperature or over night at 4°C before protein bands were detected with Super Signal^™^ West Dura Extended Duration Substrate from Pierce (Pierce Biotechnologies, Rockford, IL, USA) and visualized by the ChemiDoc MP System from Bio‐Rad Laboratories. Image Lab Software (Bio‐Rad Laboratories) was utilized for densitometric quantitations. Adobe Illustrator and Adobe Photoshop were used for image processing.

### Immunoprecipitation

For detection of HER2 ubiquitination and phosphorylation, cells were treated as described in figure legends before HER2 was precipitated under denaturing conditions as previously described 21, using anti‐HER2 antibody (clone 42) bound to Dynabeads Protein A‐coupled magnetic beads (Life Technologies) in 0.2 M NaH_2_PO_4_, 0.2 M Na_2_HPO_4_ and 0.05% Triton X‐100. For co‐immunoprecipitation analysis, cells were washed three times in ice‐cold PBS and lysed for 30 min. on ice in lysis buffer composed of 20 mM 4‐(2‐hydroxyethyl)‐1‐piperazineethanesulfonic acid (HEPES), 0.1% Triton X‐100, 2 mM MgCl_2_, 100 mM NaCl, 0.1 mM EDTA supplemented with 1:100 (v/v) of Protease and Phosphatase inhibitor cocktails (P8340 and P5726, respectively), 60 mM Octyl β‐d‐glucopyranoside and 5 mM *N*‐Ethylmaleimide. HER2 was precipitated using anti‐HER2 antibody (29D8) and Dynabeads Protein A‐coupled magnetic beads. For precipitation of GFP‐tagged CHIP, GFP‐Trap^®^_MA Kit (ChromoTek Inc., Hauppauge, NY, USA) was used and 10 μg/ml of the proteasome inhibitor MG132 was added to the lysis buffer. Proteins were eluted in 2× sample buffer 23 and the immunoprecipitated material as well as total cell lysates were subsequently subjected to immunoblotting.

### Immunoelectron microscopy

Cells were fixed with 4% methanol‐free formaldehyde and 0.1% glutaraldehyde (Electron Microscopy Sciences, Hatfield, PA, USA) in 0.2 M HEPES and prepared for cryo immunoelectron microscopy basically as described 24. Antibodies used to label for HER2 were clone TAB 250 to the extracellular domain and Ab‐3 to the intracellular domain. Bound antibodies were visualized with colloidal gold coated with protein A (G. Posthuma, Utrecht, The Netherlands). Sections were examined with a Tecnai G^2^ Spirit TEM (FEI, Eindhoven, The Netherlands) equipped with a Morada digital camera by iTEM software (Olympus Soft Imaging Solutions, Munster, Germany). Images were processed with Adobe Photoshop (Adobe Systems Inc.).

## Results

### The combination of two antibodies is needed for efficient internalization and degradation of HER2

**Figure 1:**
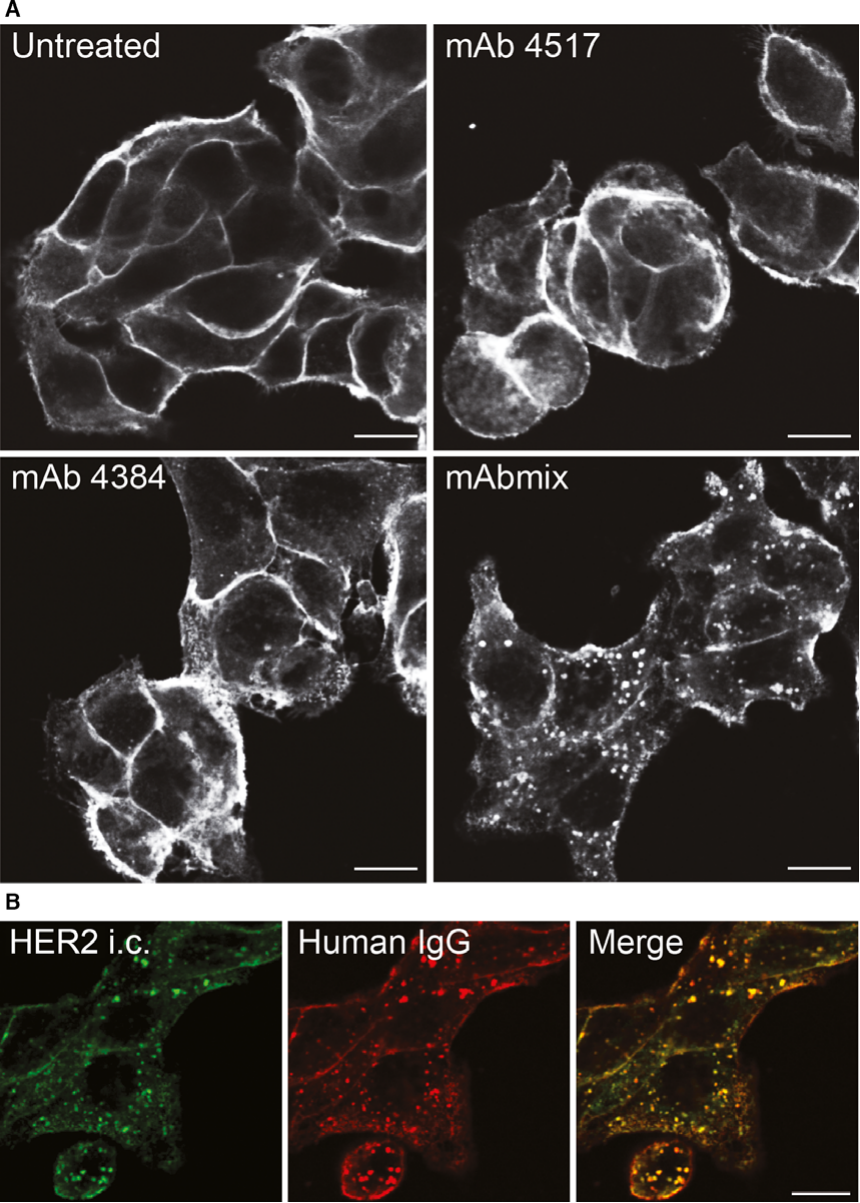
The combination of two antibodies is needed for efficient internalization of HER2. (**A**) OE19 cells were incubated in growth medium without mAbs (Untreated) or with mAb 4517, mAb 4384 or a combination of mAb 4517 and mAb 4384 (mAbmix) for 4 hrs, before the cells were fixed, permeabilized and immunostained using an antibody to the intracellular domain of HER2 (clone Z4881). (**B**) OE19 cells were incubated in growth medium containing the mAb mixture for 4 hrs. HER2 intracellular domain (HER2 i.c.) was localized as in (**A**), and the mAb mixture was localized using antibody to human IgG. Micrographs representative for three experiments are shown, scale bars: 10 μm.

**Figure 2:**
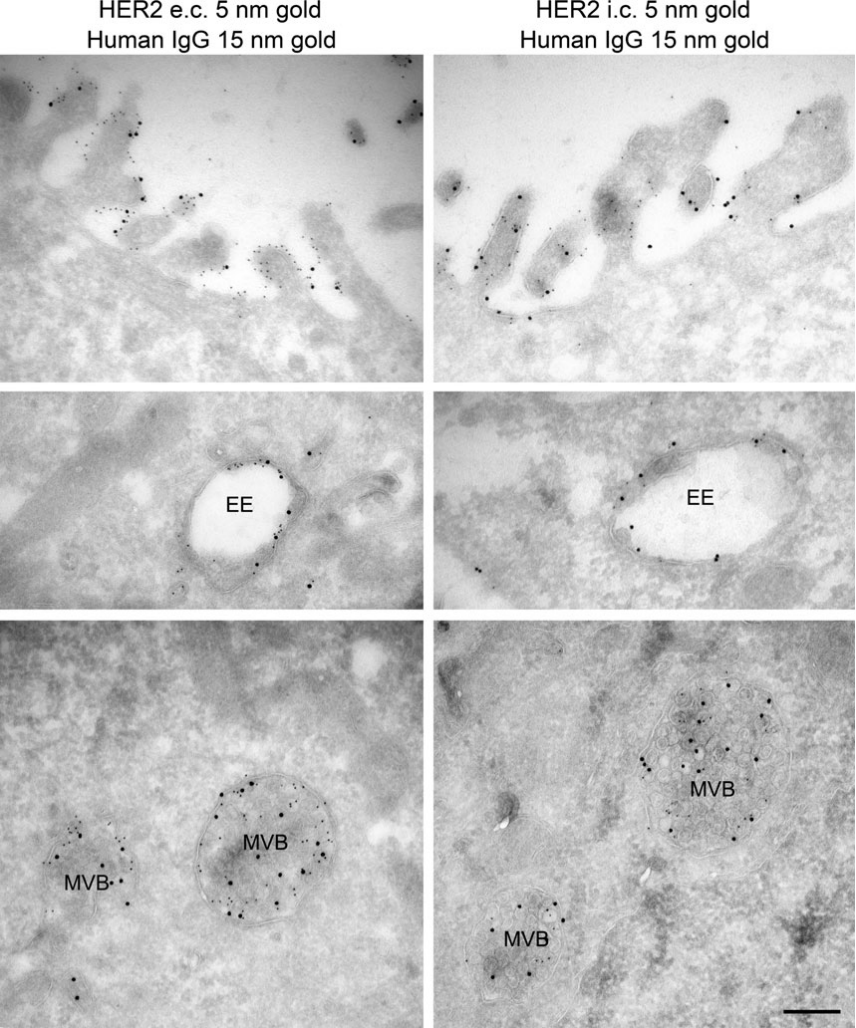
Incubation with the mAb mixture induces up‐concentration of HER2 in endosomal compartments. OE19 cells incubated in growth medium containing the mAb mixture for 4 hrs were prepared for immunoelectron microscopy and double labelled with antibodies to the extracellular (e.c.) or intracellular (i.c.) domain of HER2 (small gold particles) and antibodies to human IgG (large gold particles). The upper panels show labelling localized to the plasma membrane, the middle panels show labelling in early endosome (EE) like compartments, and the lower panels show labelling localized to multivesicular bodies (MVB), scale bar: 250 nm.

Mixtures of anti‐HER2 antibodies can cause internalization and degradation of HER2 19. To characterize these findings in more detail we chose the two antibodies mAb 4517 and mAb 4384, which bind to domains IV and III in the extracellular region of HER2, respectively. The specific pair of antibodies was chosen as they synergistically inhibited cancer cell growth *in vitro* (Fig. S2) and *in vivo* and was the two‐mAb mixture with the broadest inhibitory effect across a panel of cell lines 19, 25. Initially, we investigated to what extent the two mAbs alone or in combination induce HER2 down‐regulation. Confocal and electron microscopy analyses of the oesophageal cancer cell line OE19 showed that in cells not exposed to mAbs, HER2 was almost exclusively localized to the plasma membrane (Fig. 1A and Fig. S3). Only a highly limited amount of HER2 was associated with clathrin‐coated pits or endosomes (Fig. S3). Also upon incubation with each mAb separately, HER2 was concentrated at the plasma membrane, only very small amounts localized to vesicular compartments (Fig. 1A). However, in cells incubated with the mAb mixture, HER2 displayed a clear vesicular localization, indicating that the mAb mixture induces internalization and/or inhibits recycling of HER2 (Fig. 1A). Quantification of HER2 localizing to EEA1‐positive compartments clearly showed that more than one mAb are required to induce endosomal localization of HER2 (Fig. S4). The same effect of the mAb mixture was observed in two other HER2 overexpressing cell lines; the gastric cancer cell line NCI‐N87 and the breast cancer cell line SK‐BR‐3 (Fig. S5). Double labelling demonstrated that the mAbs co‐localized with HER2 both at the plasma membrane and in vesicular compartments with morphology resembling both early endosomes and multivesicular bodies (MVBs) (Figs 1B and 2) supporting that the mAbs are internalized in complex with HER2. Figure 2 further shows that there was no obvious mAb mixture‐induced clustering of HER2 at the plasma membrane. HER2 localization in clathrin‐coated pits did, however, appear to be more frequent (Fig. S6), although most coated pits still were negative for the mAb‐HER2 complex.

**Figure 3:**
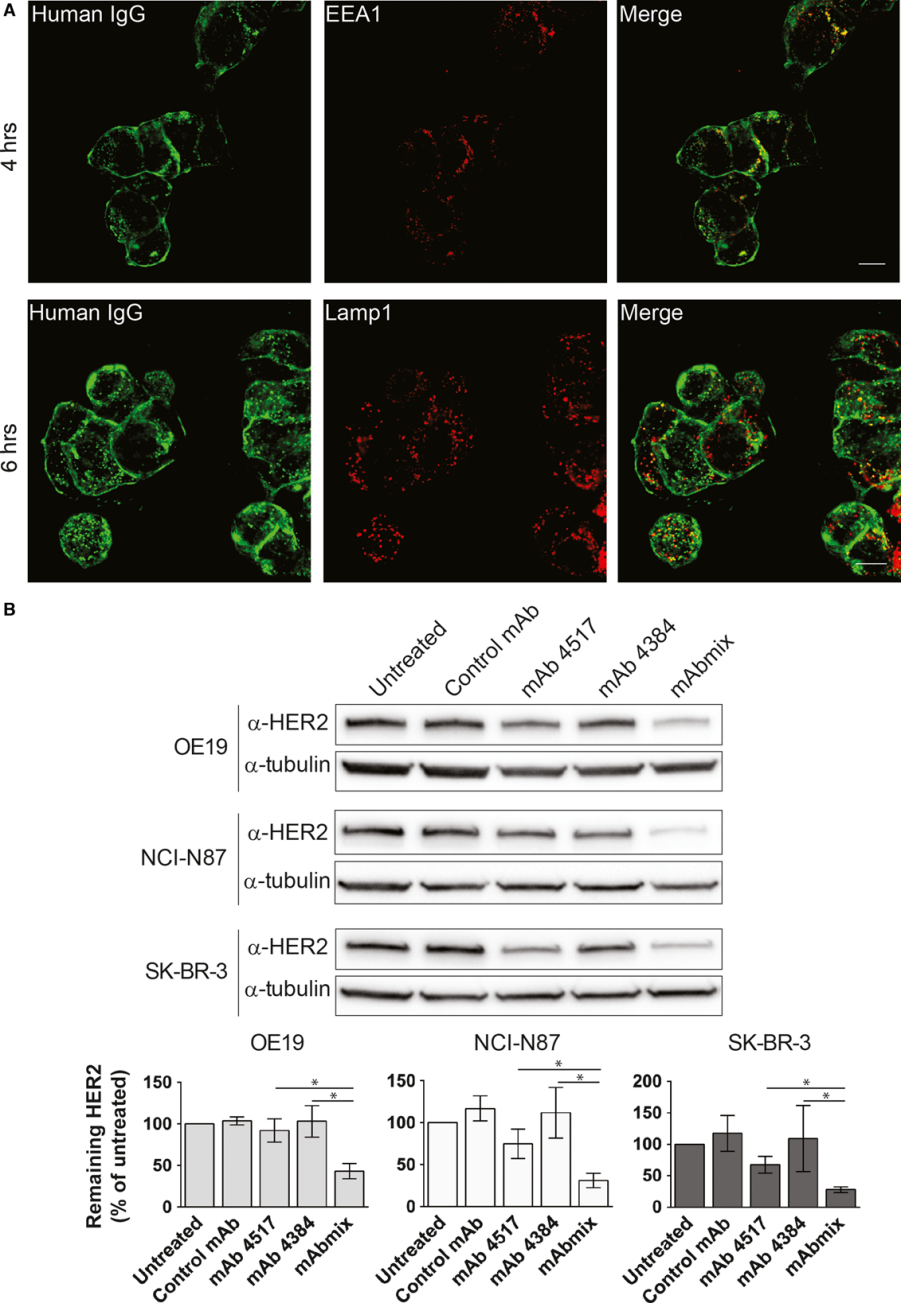
The mAb mixture induces degradation of HER2 *via* a classic endocytic degradative pathway. (**A**) OE19 cells were incubated in growth medium containing the mAb mixture for 4 or 6 hrs, before fixation, permeabilization and immunostaining with antibodies to human IgG. Early endosomes were identified by staining for EEA1 and late endosomes/lysosomes were identified by staining for Lamp1. One representative experiment out of three is shown, scale bars: 10 μm. (**B**) OE19, NCI‐N87 and SK‐BR‐3 cells were incubated in growth medium without mAbs (Untreated) or with negative control mAb, mAb 4517, mAb 4384 or the combination of mAb 4517 and mAb 4384 (mAbmix) for 24 hrs, before lysis and immunoblotting using an antibody to HER2 (clone 42). Blotting for tubulin was used as loading control. One representative experiment of three is shown. Net luminescence of the bands corresponding to HER2 was quantified and normalized to the loading control, and average values of at least three independent experiments ±S.D. were plotted as percentage of HER2 in untreated cells. Statistical analysis revealed *P* < 0.05 in all three cell lines when comparing mAbmix *versus* mAb 4517 or mAb 4384.

It is still discussed whether concentration of HER2 at the plasma membrane is caused by endocytosis resistance or a rapid recycling. Incubation with monensin, which is known to inhibit recycling, caused a slight increase in the vesicular localization of HER2 (Fig. S7), however, not as strong as upon incubation with the mAb mixture, demonstrating that the mAb mixture indeed induces internalization of HER2. Further characterization revealed that the internalized mAb mixture upon 4 hours incubation mainly localized to EEA1‐positive early endosomes (Fig. 3A upper panels and Fig. S4A). Localization in Lamp1‐positive late endosomes/lysosomes could be detected after 6 hours (Fig. 3A lower panels). Furthermore, upon incubation with mAbs for 24 hrs, the mAb mixture, but not the single mAbs, induced substantial degradation of HER2 in all the three cell lines tested (Fig. 3B). Altogether, the data demonstrate that the mAb mixture, but not single mAbs, induces HER2 internalization and degradation *via* a classic endocytic degradative pathway.

### Internalization of HER2 depends on antibody‐induced phosphorylation and ubiquitination of HER2

**Figure 4:**
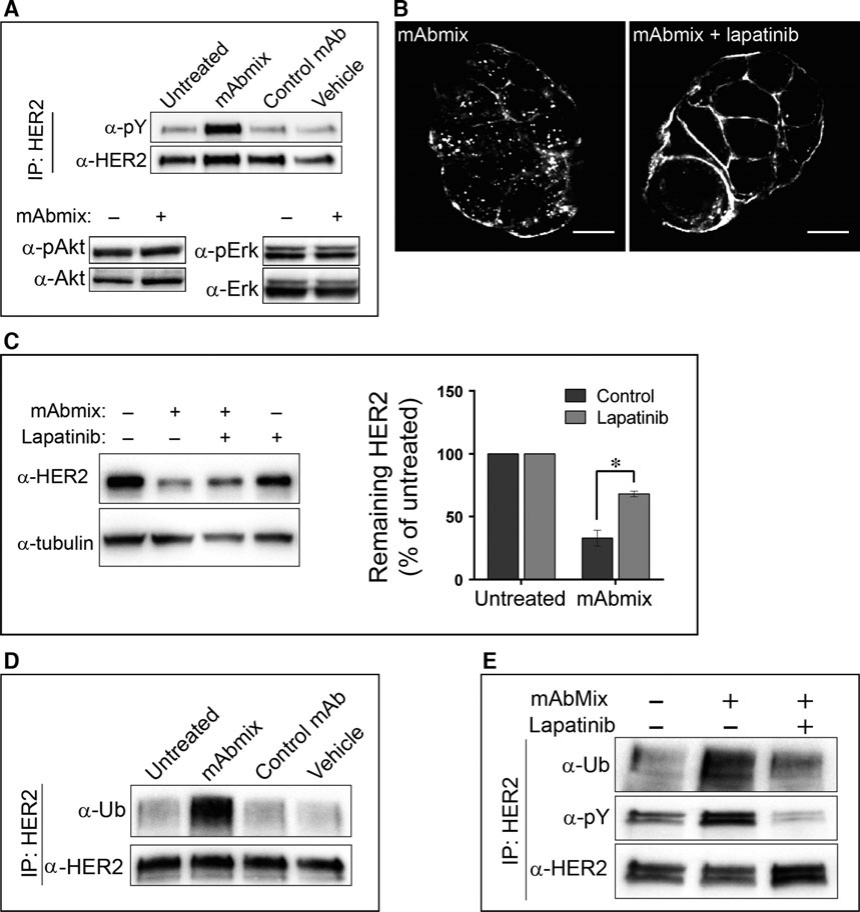
Antibody‐induced ubiquitination, internalization and degradation of HER2 depend on HER2 kinase activity. (**A**) OE19 cells were incubated in MEM only (Untreated) or in MEM with the mAb mixture, the negative control mAb or the dilution buffer for antibodies (Vehicle) for 1 hr. HER2 was immunoprecipitated and the immunoprecipitated material was analysed by immunoblotting using an antibody to HER2 (clone Z4881) before the membrane was stripped and re‐probed with an antibody to phosphorylated tyrosine (pY). Whole‐cell lysates were immunoblotted using antibodies to phosphorylated Akt (pAkt), Akt1, phosphorylated Erk1/2 (pErk) and Erk1. One representative experiment of three is shown. (**B**) OE19 cells were pre‐incubated in MEM with or without lapatinib (1 μM) for 1 hr, followed by incubation in MEM containing the mAb mixture for 4 hrs in absence or presence of lapatinib, before fixation, permeabilization and immunostaining for human IgG. Micrographs representative for three experiments are shown, scale bars: 10 μm. (**C**) OE19 cells were incubated in growth medium with the mAb mixture in absence or presence of lapatinib (1 μM) for 24 hrs, before lysis and immunoblotting using an antibody to HER2 (clone Z4881). Tubulin was used as loading control. One representative experiment of three is shown. Net luminescence of bands corresponding to HER2 was quantified and normalized to the loading control, and the average values of at least three independent experiments ±S.D. were plotted as percentage of HER2 in untreated cells. (**D**) OE19 cells were incubated in MEM only (Untreated) or in MEM with the mAb mixture, the negative control mAb or the dilution buffer for antibodies (Vehicle) for 1 hr before immunoprecipitation of HER2. The immunoprecipitates were subjected to immunoblotting using antibodies to ubiquitin (Ub). The membrane was stripped and re‐probed with an antibody to HER2 (clone Z4881). One representative experiment of three is shown. (**E**) OE19 cells were incubated in MEM only or pre‐incubated in MEM with or without lapatinib (1 μM) for 1 hr, followed by incubation in MEM with the mAb mixture with or without lapatinib for 1 hr. HER2 was immunoprecipitated and the immunoprecipitates were subjected to immunoblotting using antibodies to ubiquitin (Ub). The membrane was stripped and re‐probed with antibodies to phosphorylated tyrosine (pY) and to HER2 (clone Z4881). One representative experiment of three is shown.

Immunoprecipitation showed that the mAb mixture clearly increased the tyrosine phosphorylation of HER2 (Fig. 4A upper panel). Nevertheless, the mAb mixture did not increase phosphorylation of the downstream signalling effectors Akt and Erk (Fig. 4A lower panels). Interestingly, the dual EGFR/HER2 kinase inhibitor lapatinib 26 which inhibited the phosphorylation of HER2 (Fig. 4E) also reduced antibody‐induced internalization and degradation of HER2 (Fig. 4B and C), indicating that the HER2 kinase activity is crucial for these processes.

Although HER2 is considered to be endocytosis resistant, ubiquitination may induce endocytic down‐regulation of HER2 (reviewed in 1). It has been shown that binding of mAbs can induce HER2 ubiquitination and degradation 13, 16. In line with this, the mAb mixture used in this study induced a strong HER2 ubiquitination (Fig. 4D), but no increased ubiquitination was observed upon incubation with each mAb separately (Fig. S8). Moreover, when cells were incubated with the mAb mixture in the presence of lapatinib, the ubiquitination was clearly reduced (Fig. 4E). Based on these data, we speculate that a HER2 kinase activity‐dependent ubiquitination acts as the signal for antibody‐induced HER2 endocytosis.

**Figure 5:**
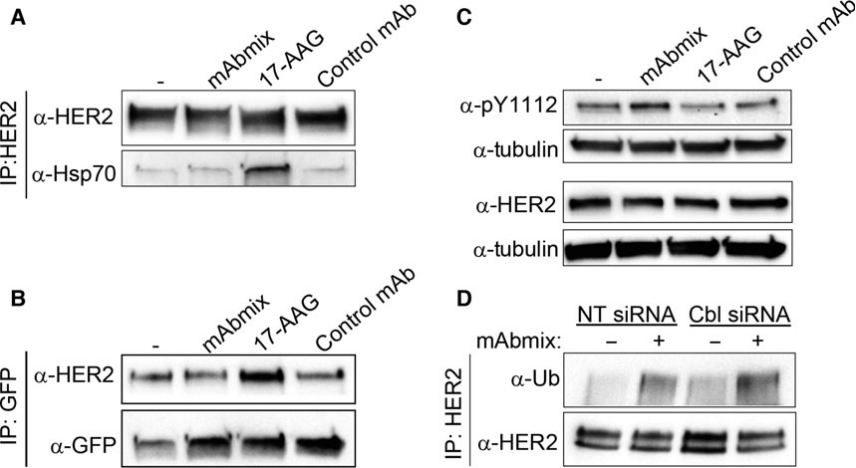
Antibody‐induced ubiquitination of HER2 is independent of both Hsp70 recruitment and the ubiquitin ligase Cbl. (**A**) OE19 cells were incubated in MEM only or in MEM with the mAb mixture, 17‐AAG (3 μM) or the negative control mAb for 1 hr, before co‐immunoprecipitation analysis using an antibody to HER2 (29D8). The immunoprecipitates were subjected to immunoblotting using antibodies to HER2 (clone 42) and to Hsp70. One representative experiment of three is shown. (**B**) OE‐19 cells were transiently transfected with plasmid‐encoding GFP‐tagged CHIP. Cells were incubated in MEM only or in MEM with the mAb mixture, 17‐AAG (3 μM) or the negative control mAb for 1 hr, before co‐immunoprecipitation analysis using GFP‐Trap^®^. The immunoprecipitated proteins and total cell lysates were analysed by immunoblotting using antibodies to HER2 (clone Z4881) and GFP. (**C**) OE19 cells were incubated in MEM only or in MEM with the mAb mixture, 17‐AAG (3 μM) or the negative control antibody for 1 hr. Cell lysates were subjected to immunoblotting using antibodies to phosphorylated tyrosine 1112 in HER2 (pY1112), HER2 (clone 42) and to tubulin (loading control). One representative experiment of three is shown. (**D**) Cbl‐b and c‐Cbl were knocked down in OE19 cells using siRNA as described in Materials and Methods. Non‐targeting (NT) siRNA was used as control. Cells were incubated in MEM with or without the mAb mixture for 1 hr, before HER2 was immunoprecipitated under denaturing conditions. The immunoprecipitates were subjected to immunoblotting using antibodies to ubiquitin (Ub). The membrane was stripped and re‐probed with antibody to HER2 (clone Z4881). One representative experiment of three is shown.

Inhibition of Hsp90 disrupts the HER2‐Hsp90 complex with simultaneous recruitment of Hsp70 27 and the ubiquitin ligases CHIP and Cullin5 which mediate ubiquitination and down‐regulation of HER2 28, 29, 30. As the mAb mixture induces HER2 ubiquitination, recruitment of Hsp70 was investigated. However, whereas the Hsp90‐inhibitor 17‐AAG induced a strong recruitment of Hsp70, no such recruitment was seen upon incubation with the mAb mixture (Fig. 5A). We further investigated whether CHIP and/or Cullin5 are recruited to HER2 upon incubation with the mAb mixture. Interaction with endogenous CHIP was not detected. We did, however, find HER2‐CHIP interaction in cells overexpressing GFP‐tagged CHIP. As shown in Figure 5B, the HER2‐CHIP interaction increased in response to 17‐AAG, but not upon incubation with the mAbs. We were not able to detect HER2 interaction with Cullin5 (endogenous or overexpressed), neither upon incubation with 17‐AAG nor with the mAb mixture (data not shown). Although the latter may represent a sensitivity problem, these results suggest that CHIP and Cullin5 are not responsible for the mAb mixture‐induced ubiquitination of HER2. We thus further investigated whether the ubiquitination is mediated by Cbl. The Cbl proteins (reviewed in 31), which are involved in ligand‐induced EGFR ubiquitination (reviewed in 32), have been reported to bind to pY 1112 in HER2 and to mediate HER2 ubiquitination and degradation upon binding of a mAb 13. Interestingly, we observed increased phosphorylation of this tyrosine residue upon incubation with the mAb mixture, but not upon incubation with 17‐AAG (Fig. 5C). Strikingly, however, siRNA‐mediated knock down of c‐Cbl and Cbl‐b, which clearly inhibited EGF‐induced EGFR ubiquitination (Fig. S9A and B), did not reduce the mAb mixture‐induced ubiquitination of HER2 (Fig. 5C).

### Antibody‐induced internalization of HER2 is dynamin dependent, but only partly depending on clathrin

**Figure 6:**
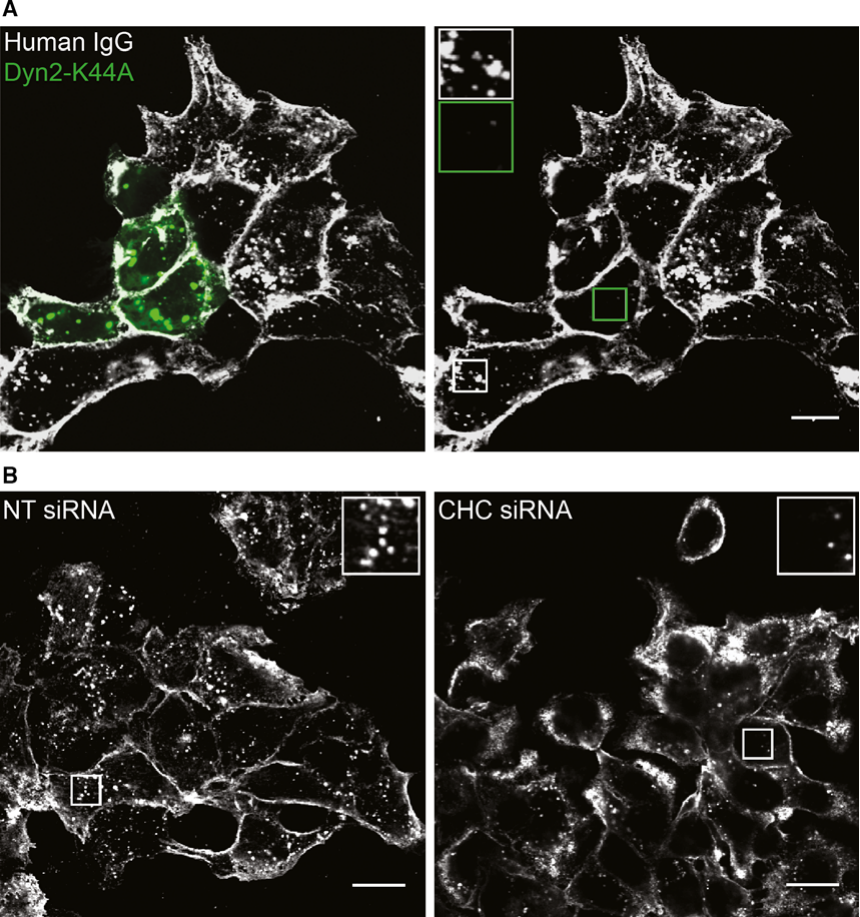
Antibody‐induced internalization of HER2 depends on dynamin 2 but only partially on clathrin. (**A**) OE19 cells were transiently transfected to express GFP‐tagged dynamin 2 K44A (Dyn2‐K44A). Cells were treated with the mAb mixture in MEM for 4 hrs, before fixation, permeabilization and immunostaining for human IgG. Micrographs representative for three experiments are shown. Left panel; overlay of Dyn2‐K44A (green) and human IgG. Right panel; human IgG only. Insets showing high magnification of the framed areas demonstrate lack of internalization in cells expressing dynamin 2 K44A (green frame), scale bar: 10 μm. (**B**) Clathrin heavy chain (CHC) was knocked down in OE19 cells by siRNA as described in Materials and Methods. Non‐targeting (NT) siRNA was used as control. Cells were incubated in MEM with the mAb mixture for 4 hrs before fixation. Upon permeabilization, the cells were immunostained using antibodies to human IgG. Insets show high magnification of the corresponding framed area in each panel. Micrographs representative for three experiments are shown, scale bars: 15 μm.

As a previous study showed dynamin‐dependent antibody‐induced HER2 internalization 17, we investigated the role of dynamin 2, utilizing a GTPase‐deficient dominant‐negative mutant (dynamin 2 K44A). Indeed, internalization of the mAb mixture‐HER2 complex was inhibited in cells expressing the mutant (Fig. 6A), confirming a role of dynamin 2 in antibody‐induced internalization of HER2. Dynamin 2 is involved in both clathrin‐dependent and various clathrin‐independent endocytic pathways 33, 34, Our immunoelectron microscopy studies showed a limited amount of the mAb–HER2 complex localizing to clathrin‐coated pits and vesicles (Fig. S6), and it has previously been reported that clathrin‐mediated endocytosis is the main pathway for Geldanamycin‐induced HER2 endocytosis 35 and for constitutive internalization of a chimeric HER2‐Ub_4_ construct 36. On the basis of this, we knocked down clathrin heavy chain to inhibit clathrin‐mediated endocytosis and found that whereas 17‐AAG‐induced internalization of HER2 was blocked (Fig. S10B and C), the vesicular localization of the mAb‐HER2 complex was reduced, but not blocked (Fig. 6B and Fig. S10D). This limited effect was confirmed in SK‐BR‐3 and NCI‐N87 cells (data not shown). These findings strongly suggest that although antibody‐induced internalization of HER2 to some degree occurs by clathrin‐mediated endocytosis, alternative pathways function in parallel.

### Antibody‐induced internalization of HER2 depends on actin polymerization

**Figure 7:**
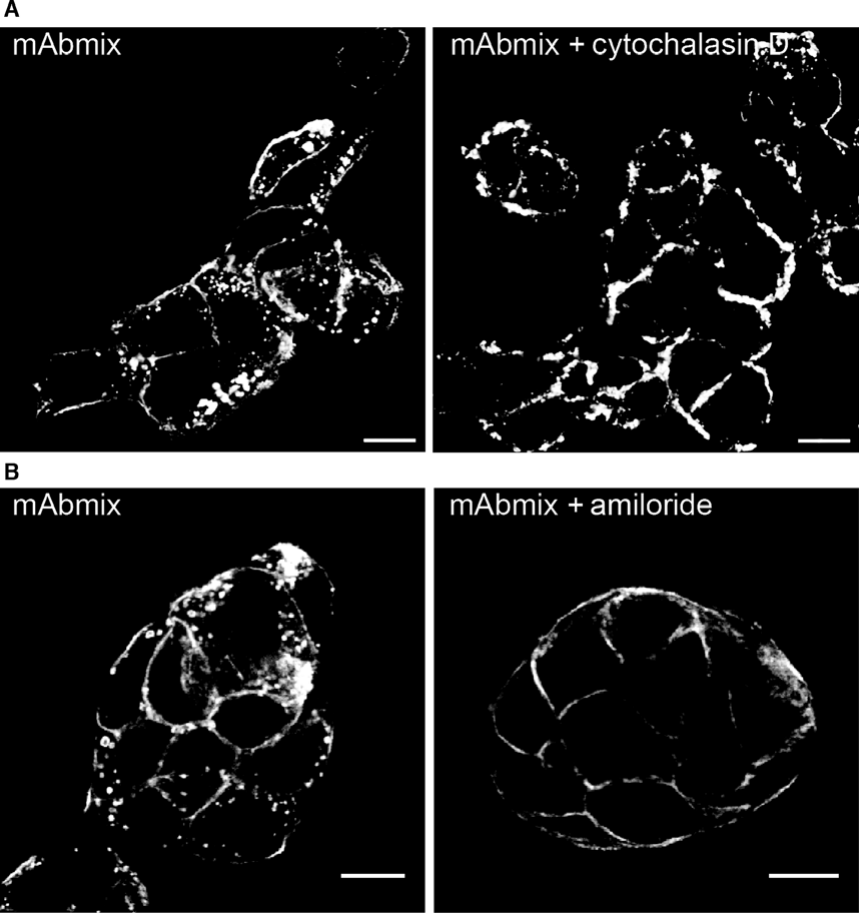
Macropinocytosis is a major pathway for antibody‐induced internalization of HER2. (**A**) OE19 cells were pre‐incubated in growth medium with or without cytochalasin D (5 μM) for 30 min. and further incubated with the mAb mixture for 4 hrs in the absence or presence of cytochalasin D. Upon fixation and permeabilization, the cells were stained for human IgG. Representative micrographs from one of three experiments are shown, scale bars: 10 μm. (**B**) OE19 cells were pre‐incubated with or without amiloride (250 μM) in MEM for 1 hr, before incubation with or without the mAb mixture in MEM for 4 hrs before fixation, permeabilization and immunostaining for human IgG (see also Fig. S12). Micrographs representative for three experiments are shown, scale bars: 10 μm.

To further investigate mechanisms underlying antibody‐induced HER2 internalization, we studied the involvement of functional actin and macropinocytosis. Macropinocytosis is an important pathway for internalization of larger areas of plasma membrane, the internalization depends on actin and both dynamin‐dependent and ‐independent forms of macropinocytosis are reported (reviewed in 37). To investigate this, cells were incubated with cytochalasin D which inhibits actin polymerization 38, or amiloride which is used as a macropinocytosis inhibitor 39. When cells were exposed to cytochalasin D, actin filaments were disrupted (Fig. S11) and the mAb mixture‐induced internalization of HER2 was strongly inhibited (Fig. 7A). Also amiloride almost completely inhibited HER2 internalization (Fig. 7B). As amiloride potentially can affect clathrin‐mediated endocytosis 40, transferrin was used as control, and only a minor effect on transferrin internalization was observed (Fig. S12). Altogether these data demonstrate that antibody‐induced internalization of HER2 occurs through a macropinocytosis‐like pathway that involves dynamin.

## Discussion

Although most studies conclude that HER2 is resistant to internalization, it has been reported that HER2 has a basal, slow internalization followed by efficient recycling (reviewed in 1), which is in line with the weak, but constitutive, endosomal localization we observe for HER2. Furthermore, it was reported that trastuzumab did not induce HER2 internalization or degradation, but instead was internalized and recycled along with HER2 41, supporting the lack of effect we observed upon incubation with individual mAbs. This could further indicate that the effect of the mAb mixture is not to induce HER2 internalization, but rather, as suggested for antibody‐induced down‐regulation of EGFR, to inhibit its recycling 17, 20. However, compared to the effect of monensin, incubation with the mAb mixture resulted in a much stronger endosomal localization of HER2, supporting the view that a major initial effect of the mAb mixture is to induce HER2 internalization. Still, this does not exclude that antibody‐induced cross‐linking of HER2 also inhibits recycling and/or increases sorting towards late endosomes. The fate of the internalized mAbs was not studied in detail. Antigen–antibody interactions are pH sensitive, and the mAbs might dissociate from HER2 in endosomes and recycle back to the medium. Electron microscopy did, however, show co‐localization of HER2 and mAbs on internal vesicles of MVBs (see Fig. 2), suggesting that the internalized mAbs to a large extent are sorted and degraded along with HER2.

A possible signal both for internalization and endosomal sorting is ubiquitination. In line with our current results, it has previously been shown that incubation with anti‐HER2 antibodies can induce HER2 ubiquitination 13, 14, 15, 16. It is also known that Geldanamycin induces ubiquitination, internalization and degradation of HER2. Whether ubiquitination then serves as an internalization signal or as a signal for cleavage or degradation of the cytoplasmic tail of HER2, removing retention signals and/or exposing otherwise hidden internalization signals remains unclear 35, 42, 43. It has also been reported that antibodies can induce internalization of a HER2 mutant lacking both the transmembrane domain and the cytoplasmic tail, only membrane anchored by a glycosylphosphatidylinositol lipid anchor 17. This could indicate that clustering of HER2 in specific membrane domains like lipid rafts is sufficient for internalization. Our results showing that the HER2 kinase activity is required for efficient antibody‐induced internalization rule out that antibody‐induced cross‐linking is sufficient for internalization of the intact HER2, but do not exclude that a kinase‐dependent ubiquitination followed by cleavage/degradation of the cytoplasmic tail is required. Labelling for the C‐terminal part of HER2 on internal vesicles of MVBs (see Fig. 2) does, however, show that the cytoplasmic tail of HER2 is intact upon antibody‐induced internalization and endosomal sorting of HER2. This seems to rule out that cleavage is required, and favour the idea that ubiquitination can serve as internalization signal, and possibly later as signal for sorting into MVBs (reviewed in 44) followed by lysosomal degradation of HER2.

In line with Friedman *et al*. 17, we found that antibody‐induced internalization of HER2 is dynamin dependent. Dynamin is needed for clathrin‐mediated endocytosis, but as clathrin knock down only partially inhibited antibody‐induced internalization of HER2, clathrin‐mediated endocytosis is not the major pathway. Furthermore, we found that functional actin plays a central role, and that the internalization was strongly inhibited by amiloride. In line with this we have previously shown that a combination of antibodies which cross‐links the EGFR, down‐regulated the receptor by macropinocytosis 21. We thus conclude that the mAb mixture can induce internalization of HER2 *via* multiple pathways, probably operating in parallel, and suggest that in addition to clathrin‐mediated endocytosis a specific form of macropinocytosis which depends on dynamin (reviewed in 37) is involved.

The findings that the mAb mixture induces HER2 phosphorylation and that lapatinib inhibits this as well as ubiquitination and down‐regulation of HER2 are fascinating. Phosphorylation of ErbB proteins occurs by trans‐phosphorylation 45. The mAb mixture must thus either stabilize HER2‐containing dimers in an active state, or cross‐link HER2 in a way that allows the asymmetric configuration of the cytoplasmic tails needed for phosphorylation to occur. The inhibited ubiquitination seen upon incubation with lapatinib strongly suggests that tyrosine phosphorylation of HER2 is required for recruitment of the ubiquitin ligase(s). In agreement with a previous study 13, we found that the mAb mixture did induce phosphorylation of tyrosine 1112 in HER2. HER2 pY1112 corresponds to EGFR pY1045 which is the direct biding site for c‐Cbl in EGFR. However, whereas Klapper and co‐workers found that c‐Cbl was needed for HER2 ubiquitination 13, we found no effect of knocking down c‐Cbl and Cbl‐b. Although this does not rule out Cbl as an ubiquitin ligase for HER2, it suggests that other yet unidentified ubiquitin ligases are involved in the mAb mixture‐induced HER2 ubiquitination.

Pharmaceutically, an important effect of some receptor‐specific mAbs is induction of ADCC and/or complement‐dependent cytotoxicity. The finding that lapatinib, which is approved for treatment of breast cancer, appears to have opposing effects and inhibits mAb‐induced HER2 internalization, opens for different strategies in antibody‐based cancer treatment. Although treatment with mAbs only can be expected to target cells overexpressing HER2 and induce receptor down‐regulation, treatment with mAbs in combination with lapatinib will inhibit mAb‐induced down‐regulation of HER2. HER2 will, however, be kinase inactivated and the accumulation of mAb–HER2 complexes at the plasma membrane can be expected to cause an increase in ADCC as previously shown for lapatinib in combination with trastuzumab 14.

In conclusion, our data show that the combination of two anti‐HER2 antibodies against non‐overlapping epitopes induces internalization of HER2 by multiple internalization pathways. The internalization eventually results in efficient lysosomal degradation of HER2, which in tumours driven by HER2 homo‐ or heterodimers most likely will give therapy with antibody mixtures an advantage compared to therapy with single monoclonal antibodies.

## Conflict of interest

M.M.G. and M.W.P. are employed by Symphogen A/S.

## Supporting information


**Figure S1** The negative control mAb does not bind to HER2.
**Figure S2** Dose‐response curves showing the anti‐proliferative effect of mAb 4384, mAb 4517 and mAb mixture on the cancer cell lines OE19 and NCI‐N87.
**Figure S3** Localization of HER2 in untreated OE19 cells.
**Figure S4** More than one mAb are needed for efficient internalization of HER2.
**Figure S5** The mAb mixture induces internalization of HER2 in NCI‐N87 and SK‐BR‐3 cells.
**Figure S6** Coated pit localization of the mAb‐HER2 complex.
**Figure S7** The mAb mixture‐induced endosomal localization of HER2 is as a result of increased internalization rather than inhibited recycling.
**Figure S8** The mAb mixture, but not single mAbs, induces ubiquitination of HER2.
**Figure S9** Knock down of Cbl‐b and c‐Cbl inhibits EGF‐induced ubiquitination of EGFR.
**Figure S10** Knock down of clathrin heavy chain differently affects 17‐AAG‐ and mAb mixture‐induced internalization of HER2.
**Figure S11** Cytochalasin D disrupts actin filaments.
**Figure S12** Amiloride, which inhibits antibody‐induced internalization of HER2, does not inhibit clathrin‐mediated endocytosis.
