# Supplementary material for: A combination of two antibodies recognizing non‐overlapping epitopes of HER2 induces kinase activity‐dependent internalization of HER2
Source: J Cell Mol Med. 2016 Jul 28;20(10):1999–2011. doi: 10.1111/jcmm.12899 (PMC5020627; doi:10.1111/jcmm.12899)
Supplement: Supplementary file 1 — Figure S1 The negative control mAb does not bind to HER2. Figure S2 Dose‐response curves showing the anti‐proliferative effect of mAb 4384, mAb 4517 and mAb mixture on the cancer cell lines OE19 and NCI‐N87. Figure S3 Localization of HER2 in untreated OE19 cells. Figure S4 More than one mAb are needed for efficient internalization of HER2. Figure S5 The mAb mixture induces internalization of HER2 in NCI‐N87 and SK‐BR‐3 cells. Figure S6 Coated pit localization of the mAb‐HER2 complex. Figure S7 The mAb mixture‐induced endosomal localization of HER2 is as a result of increased internalization rather than inhibited recycling. Figure S8 The mAb mixture, but not single mAbs, induces ubiquitination of HER2. Figure S9 Knock down of Cbl‐b and c‐Cbl inhibits EGF‐induced ubiquitination of EGFR. Figure S10 Knock down of clathrin heavy chain differently affects 17‐AAG‐ and mAb mixture‐induced internalization of HER2. Figure S11 Cytochalasin D disrupts actin filaments. Figure S12 Amiloride, which inhibits antibody‐induced internalization of HER2, does not inhibit clathrin‐mediated endocytosis. [file JCMM-20-1999-s001.pdf]

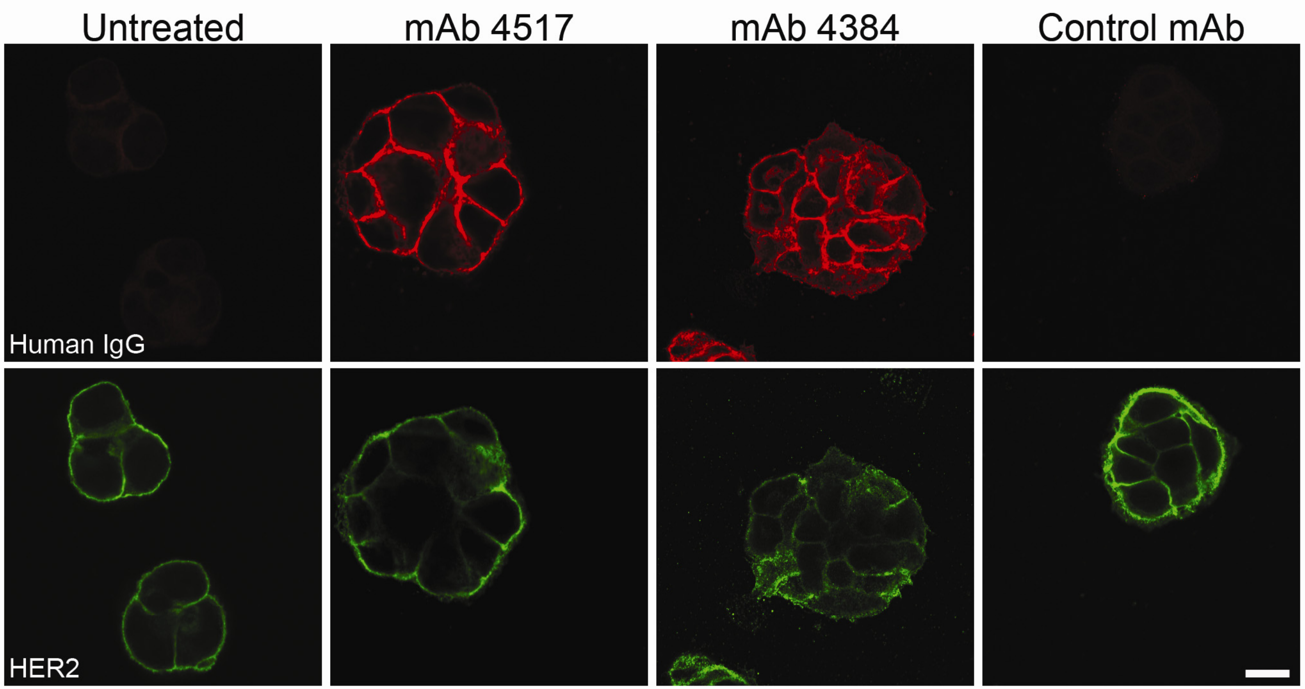

Figure S1. The negative control mAb does not bind to HER2. OE19 cells were incubated in growth medium without mAbs (Untreated) or with mAb 4517, mAb 4384 or the control mAb for 15min, before the cells were fixed, permeabilized and immunostained using an antibody to the intracellular domain of HER2 (clone Ab-3). The mAbs were localized using antibody to human IgG. Micrographs representative for three experiments are shown. Scale bar, 10 µm.

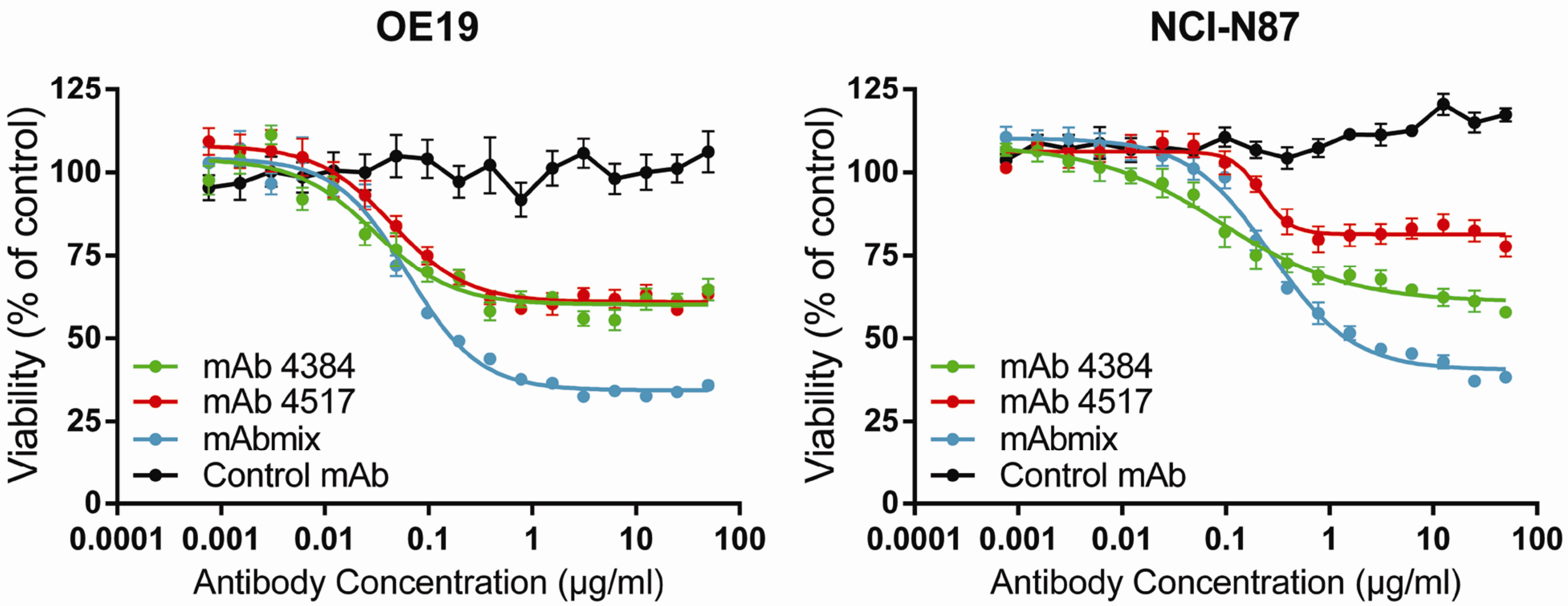

Figure S2. Dose-response curves showing the anti-proliferative effect of mAb 4384, mAb 4517 and mAb mixture on the cancer cell lines OE19 and NCI-N87. Control mAb, mAb 4384, mAb 4517 and a mixture of mAb 4384 and mAb4517 (mAbmix) were titrated 2-fold from 50 µg/ml in growth media with 2% FBS. Cells in growth media with 2% FBS were then added to the antibodies and incubated for 4 days. The viability was determined by addition of the cell proliferation reagent WST-1. The absorbance was measured at 450 nm and 620 nm (reference wavelength) using an ELISA reader. Absorbance at 620 nm was subtracted from the absorbance at 450 nm (=OD). The amount of metabolically active cells (MAC) was calculated as a percentage of the untreated control as follows:

$$\%MAC = \left(\frac{ODexp. - ODMedia}{ODuntreat. - ODMedia}\right) \times 100$$

Data are representative of three independent experiments and represented as means ± SEM.

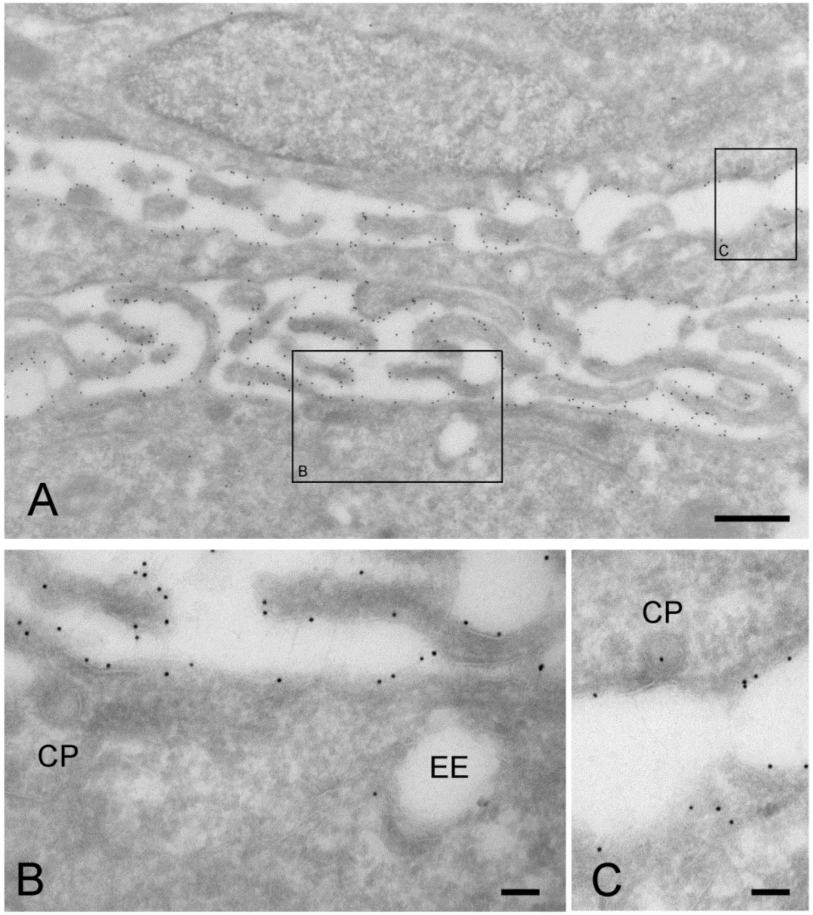

Figure S3. Localization of HER2 in untreated OE-19 cells. OE19 cells were prepared for immuno-electron microscopy and labeled with an antibody to HER2 (clone TAB250). **B** and **C** show high magnifications of the correspondingly framed areas in **A**. CP: Coated pit. EE: Early endosome. Scale bar in **A**, 500 nm, bars in **B** and **C**, 100 nm.

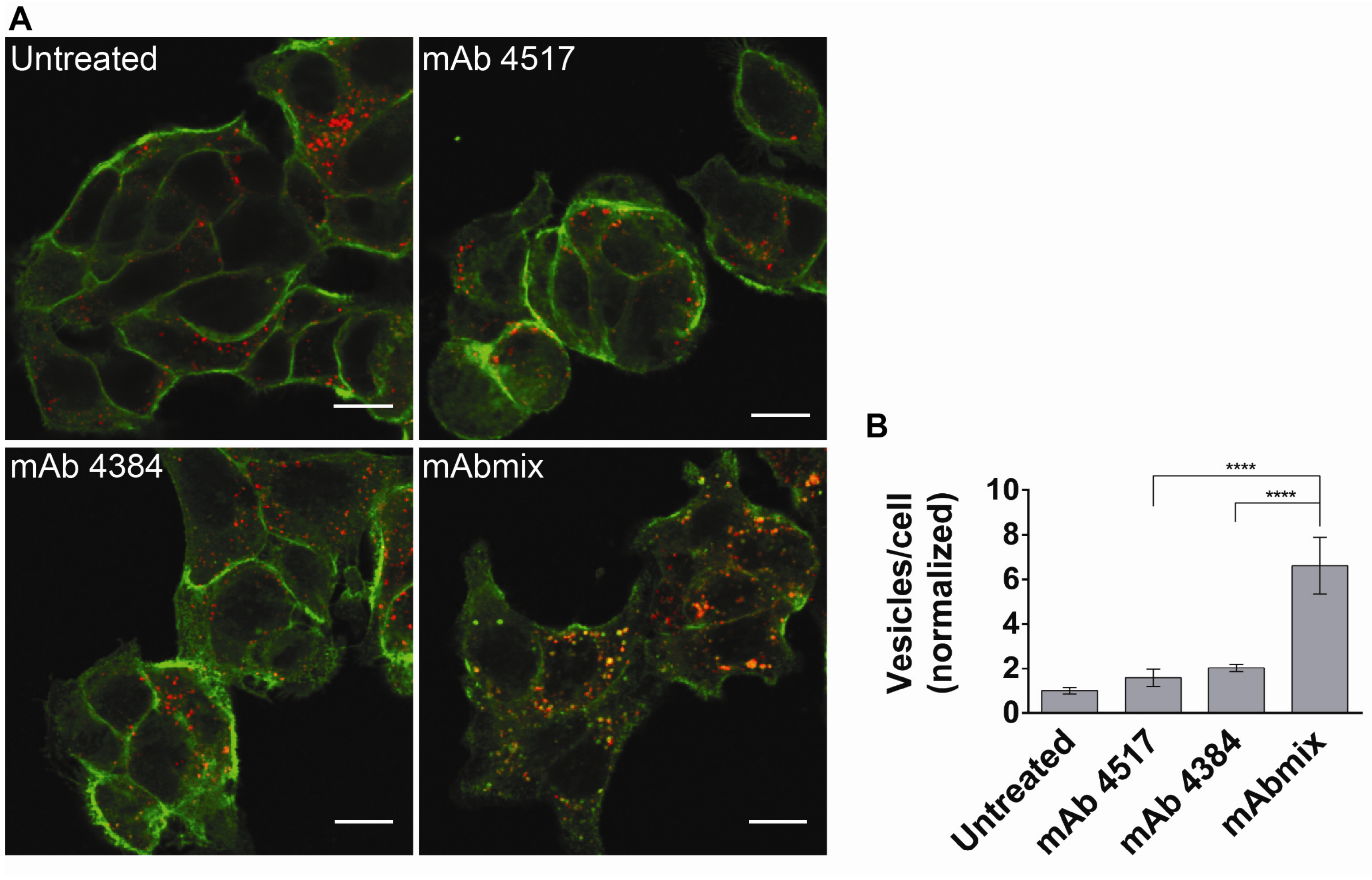

Figure S4. More than one mAb are needed for efficient internalization of HER2. (A) Labeling for HER2 (green) and EEA1 (red) (same cells as shown in Figure 1A) demonstrates mAbmix induced localization of HER2 in EEA1 positive endosomes **(B)** Vesicles positive for both EEA1 and HER2 were detected and quantified using the coloc module before spot detection function in Imaris image analysis software (Bitplane). 3 experiments were performed, the average number of cells analyzed per condition were in the range 73-122. For each experiment, the number of double positive vesicles per cell was normalized to the mean number of double positive vesicles per cell in the untreated condition, which was set to 1. Data from the 3 independent experiments were pooled and are presented as mean ± SD. Statistical analysis were performed in GraphPad Prism and revealed p-values <0.0001 when comparing mAbmix versus mAb 4517 or mAb 4384.

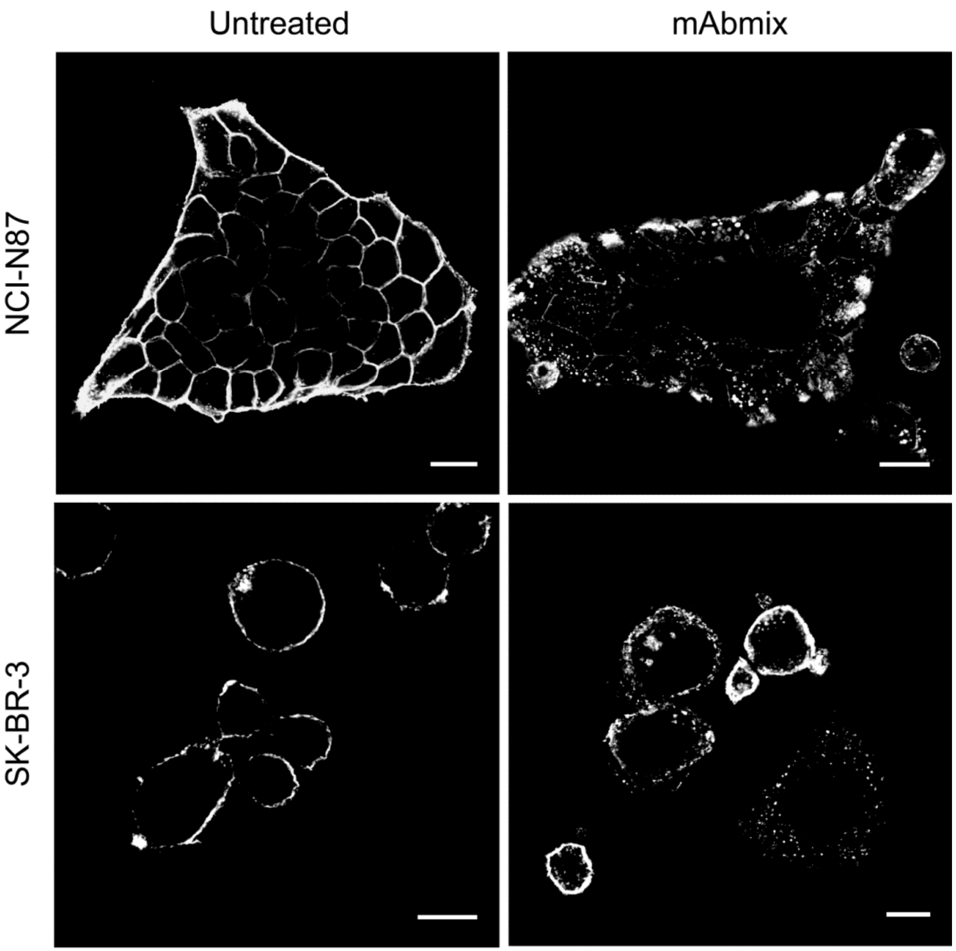

Figure S5. mAb mixture induces internalization of HER2 in NCI-N87 and SK-BR-3 cells. NCI-N87 (upper panels) and SK-BR-3 (lower panels) cells were incubated in growth medium with or without the mAb mixture for 4 h before fixation. Upon permeabilization, the cells were stained with an antibody to HER2 (clone TAB250). One representative experiment out of three is shown. Scale bars, 15 µm.

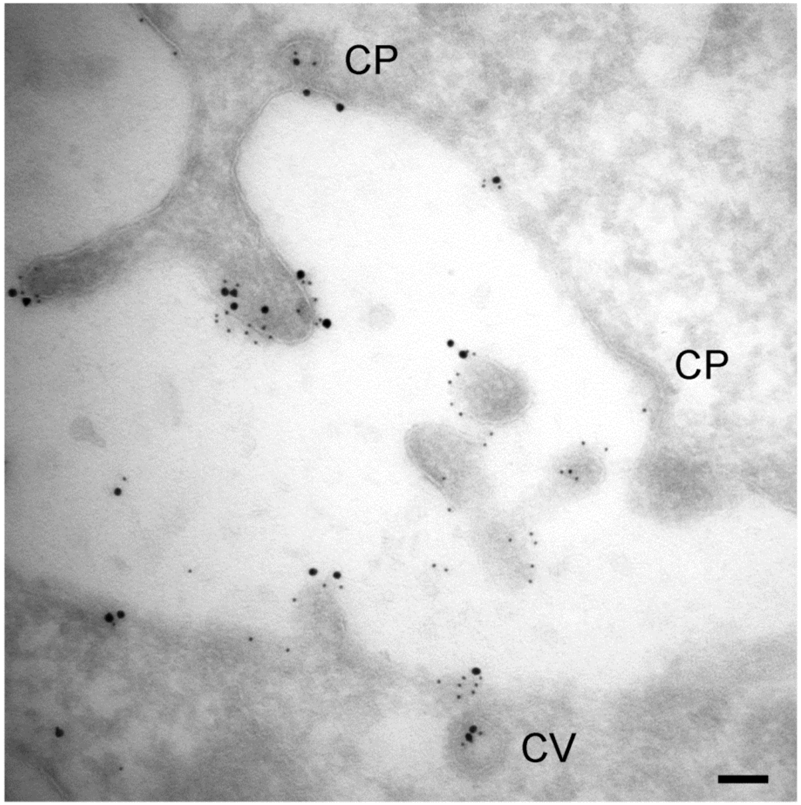

Figure S6. Coated pit localization of the mAb-HER2 complex. OE19 cells incubated with the mAb mixture for 4 h were prepared for immuno-electron microscopy and double labeled with antibodies to the extracellular domain of HER2 (clone TAB250, small gold particles) and antibodies to human IgG (large gold particles). CP: Coated pit. CV: Coated vesicle. Scale bar, 100 nm.

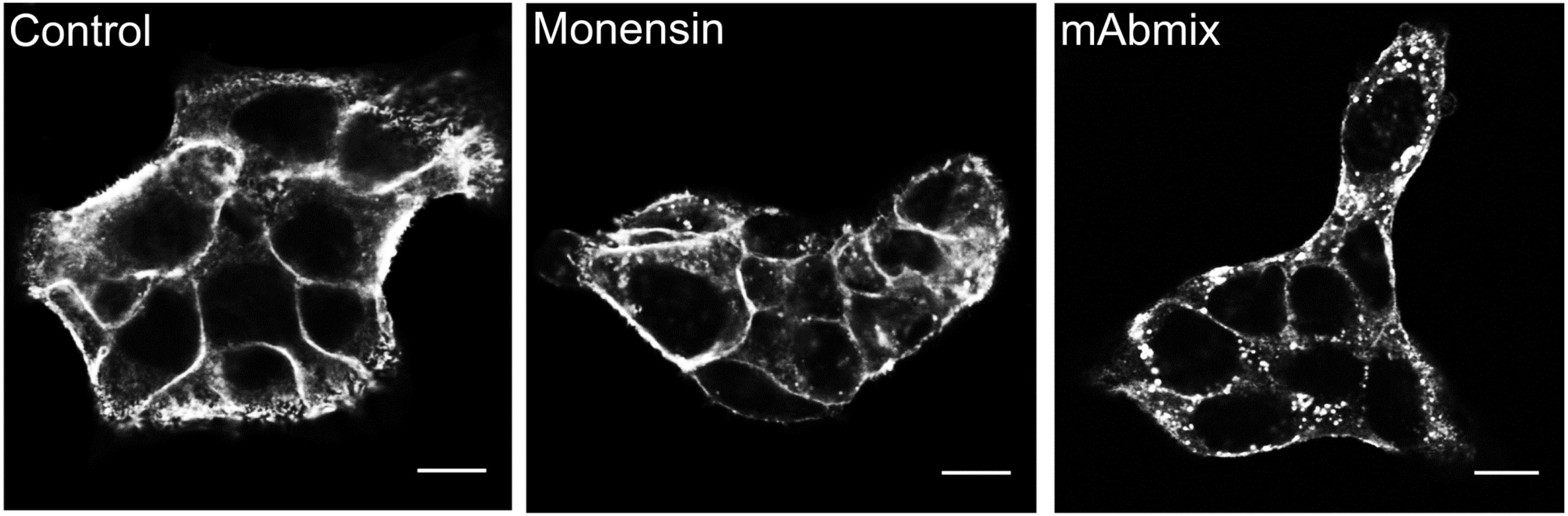

Figure S7. The mAb mixture-induced endosomal localization of HER2 is due to increased internalization rather than inhibited recycling. OE19 cells were incubated in pure growth medium (Control) or in growth medium with either monensin (50 µM), or the mAb mixture for 4 h, before fixation, permeabilization and immunostaining with antibodies to HER2 (clone Z4881). Micrographs representative for three experiments are shown. Scale bars, 10 µm.

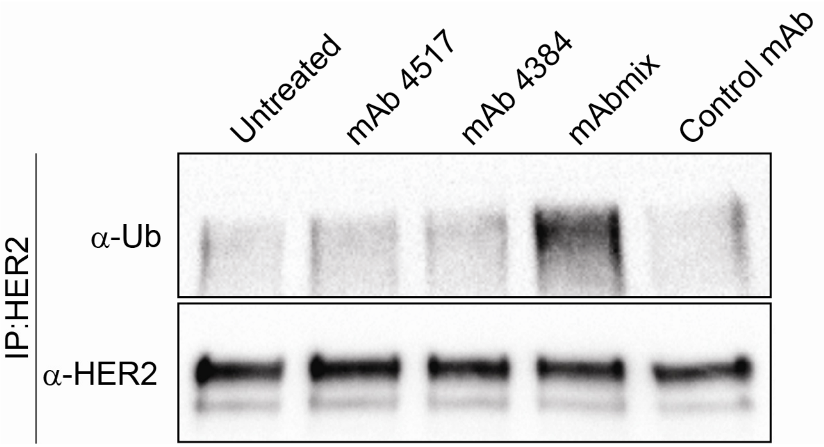

Figure S8. The mAb mixture, but not single mAbs, induces ubiquitination of HER2. OE19 cells were incubated in MEM only (Untreated) or in MEM with mAb 4517, mAb 4384, a mixture of mAb 4384 and mAb 4517 (mAbmix), or the negative control mAb for 1 h before immunoprecipitation of HER2 under denaturing conditions. The immunoprecipitates were subjected to immunoblotting using antibodies to ubiquitin (Ub). The membrane was stripped and re-probed with an antibody to HER2 (clone Z4881). One representative experiment out of three is shown.

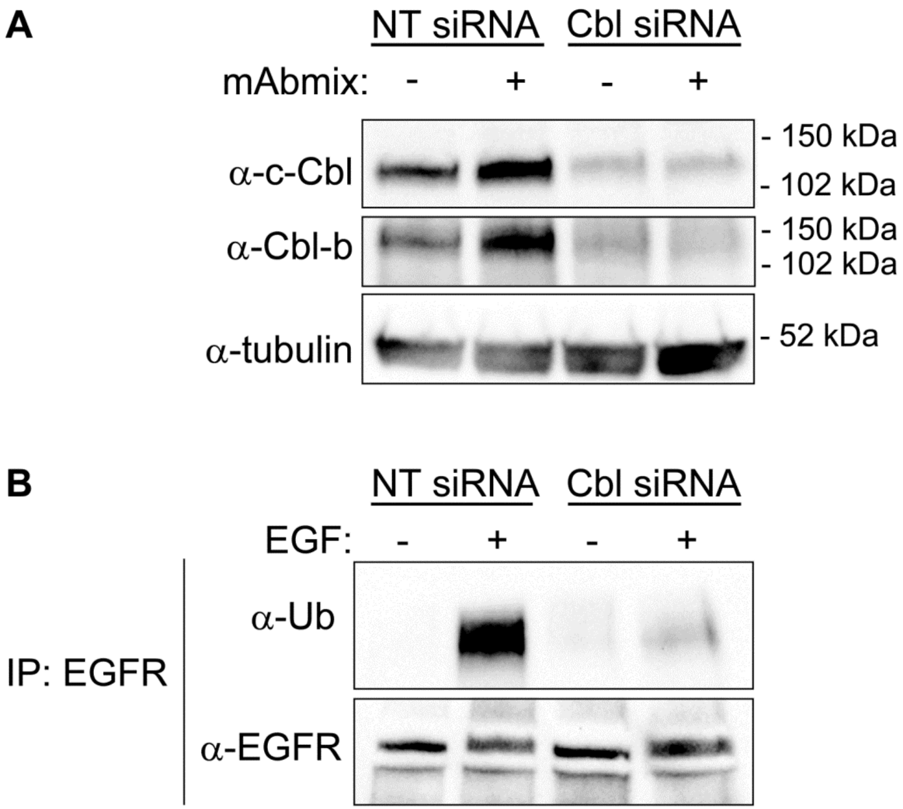

Figure S9. Knock-down of Cbl-b and c-Cbl inhibits EGF-induced ubiquitination of EGFR. Cbl-b and c-Cbl were knocked down in OE19 cells using siRNA as described in Materials and Methods. Non-targeting (NT) siRNA was used as control. **(A)** To validate the knock-down efficiency, cell lysates were initially immunoblotted using an antibody to Cbl-b (H-454, Santa Cruz Biotechnology, Inc.). The membrane was then stripped and re-probed with an antibody to c-Cbl (C-15, Santa Cruz Biotechnology, Inc.). Tubulin was used as loading control. **(B)** Cells were incubated in MEM without or with 60 ng/ml EGF (Bachem AG) for 1 h, before EGFR was immunoprecipitated under denaturing conditions using a sheep anti-EGFR antibody (Fitzgerald Industries International, Inc). The immunoprecipitates were subjected to immunoblotting using antibodies to ubiquitin (Ub). The membrane was stripped and re-probed with an antibody to EGFR. One representative experiment out of three is shown.

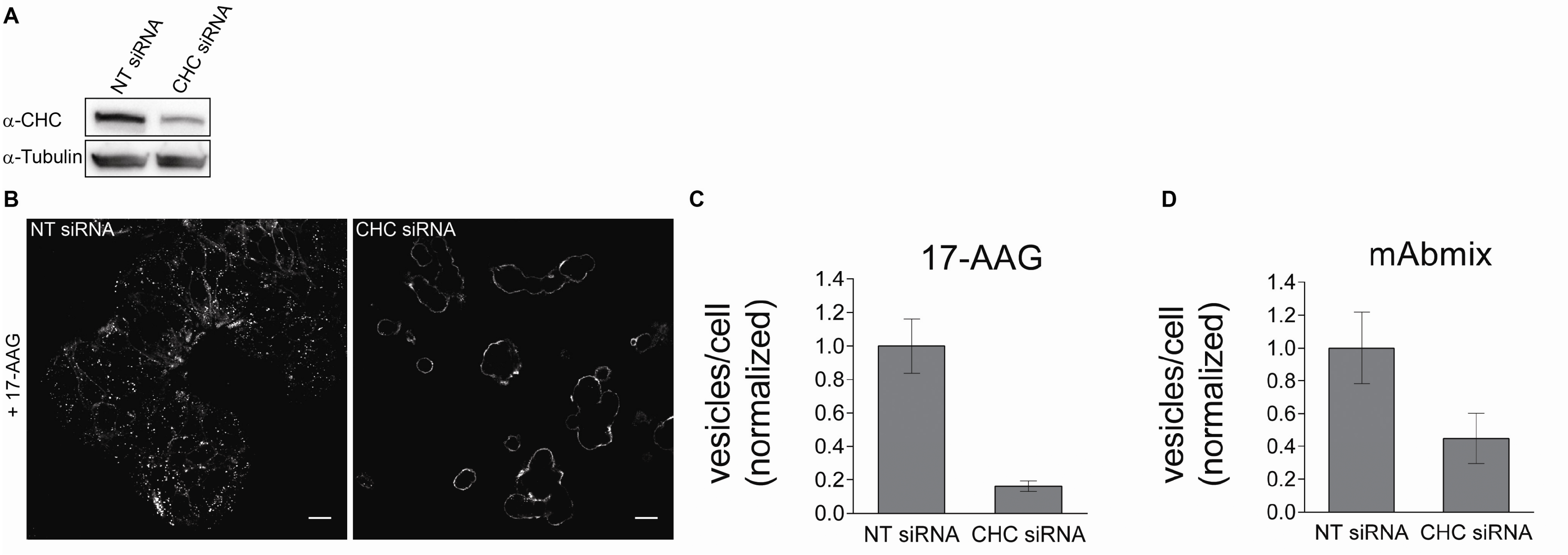

Figure S10. Knock-down of clathrin heavy chain differently affects 17-AAG- and mAb mixture-induced internalization of HER2. Clathrin heavy chain (CHC) was down-regulated in OE19 cells by siRNA. Non-targeting (NT) siRNA was used as control. **(A)** To validate the knock-down efficiency, cell lysates were immunoblotted using a mouse-anti CHC antibody (BD Biosciences). Tubulin was used as loading control. **(B)** Cells transfected with the indicated siRNA, were incubated in MEM with 17-AAG (3 µM) for 4 h before fixation. Upon permeablization, the cells were immunostained using antibodies to HER2 (clone Z4881). Scale bars, 15 µm. **(C-D)** In micrographs from the experiment described in (B) and Figure 6B, vesicles positive for both EEA1 and mAbs were detected and quantified using the coloc module before spot detection function in Imaris image analysis software (Bitplane). **(C)** Data from 1 representative experiment were analysed and for each condition (NT siRNA or CHC siRNA), 158 cells were analyzed. The number of double positive vesicles per cell was normalized to the mean number of double positive vesicles per cell in cells transfected with NT siRNA, which was set to 1. Data are presented as mean ± SD. **(D)** Data from 3 independent experiment were analysed. In each experiment an average of 87 and 158 cells were analyzed for cells transfected with NT siRNA or CHC siRNA, respectively, and the number of double positive vesicles per cell was normalized to the mean number of double positive vesicles per cell in cells transfected with NT siRNA, which was set to 1. Data from the 3 independent experiments were pooled and are presented as mean ± SD.

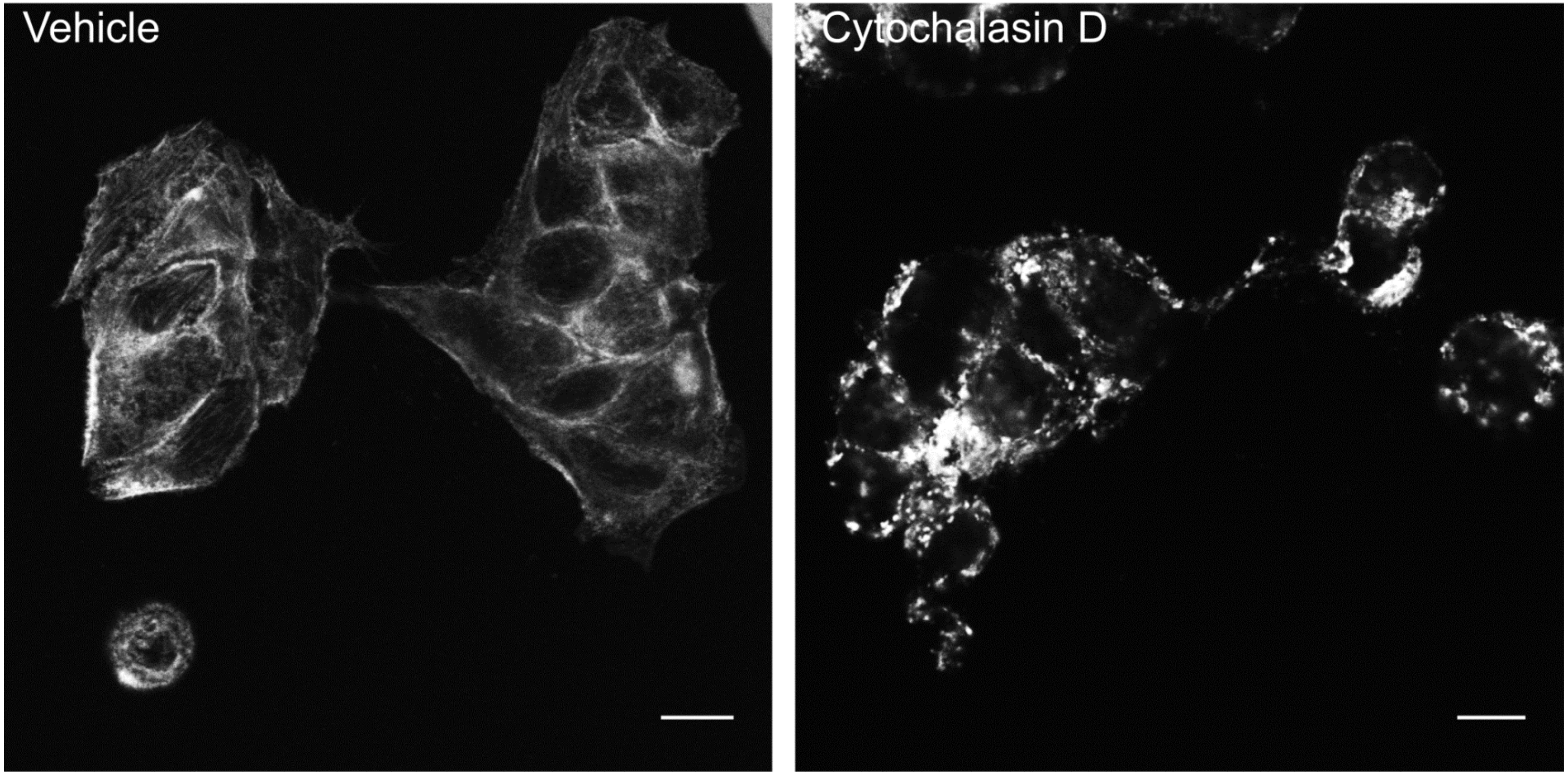

Figure S11. Cytochalasin D disrupts actin filaments. OE19 cells were incubated in growth medium without or with cytochalasin D (5 µM) for 4.5 h. Upon fixation in 4% methanol-free PFA (Ultra Pure EM Grade from Polysciences, Inc.) and permeabilization, the cells were incubated with 33 nM Alexa Fluor 555-conjugated Phalloidin (Life Technologies) in PBS for 30 min at RT. Micrographs representative for three experiments are shown. Scale bars, 10 µm.

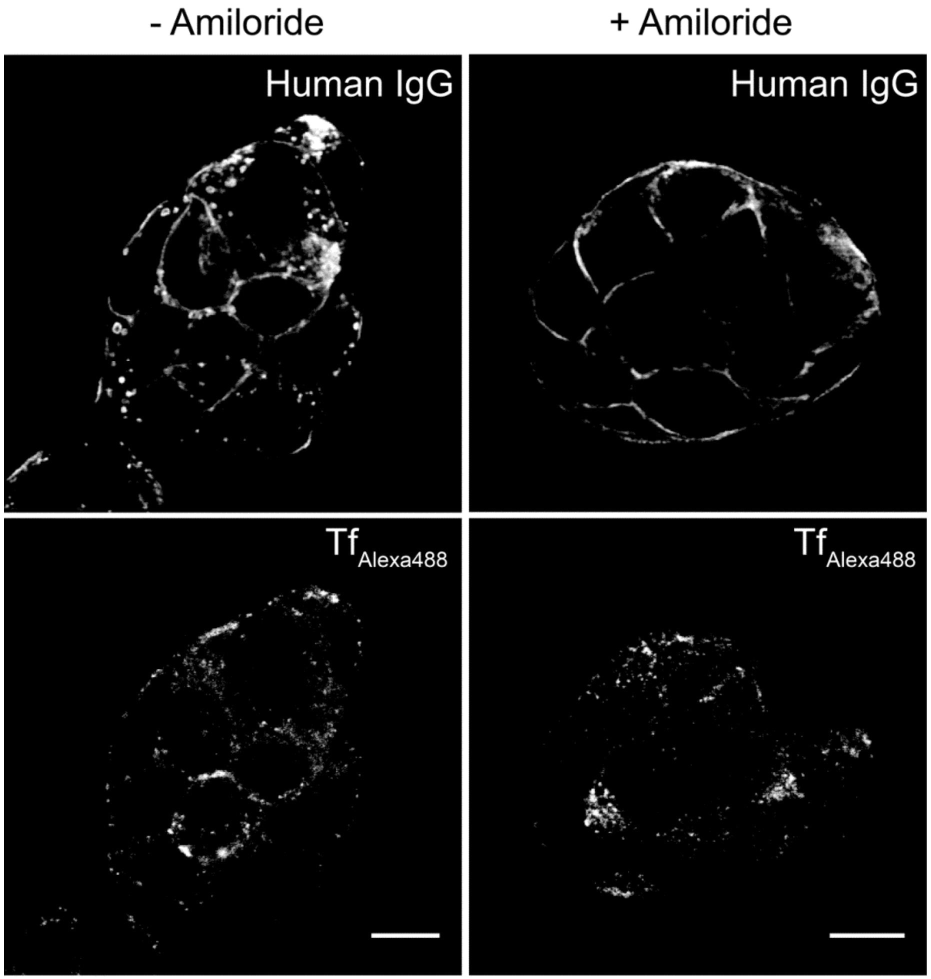

Figure S12. Amiloride which inhibits antibody-induced internalization of HER2, does not inhibit clathrin mediated endocytosis. OE19 cells were pre-incubated with or without amiloride (250 µM) in MEM for 1 h, before incubation with or without the mAb mixture in MEM for 4 h. 20 µg/ml Alexa 488-conjugated transferrin (Life Technologies) was added to the cells the last 15 min before fixation, permeabilization and immunostaining for human IgG. The micrographs show the same cells as presented in Figure 7. Scale bars, 10 µm.
